# Medical and Philosophical News

**Published:** 1781-05

**Authors:** 


### SECTION III.

### Medical and Philosophical News.

AN academy of ſciences has lately been founded at Batavia in the Eaſt Indies. It conſiſts of a preſident, a director, and an unlimited number of members. The principal objects of this new inſtitution are, Commerce, and Agriculture; but beſides theſe, it means to extend its views to Phyſic, Natural Philoſophy, Natural Hiſtory, Antiquities, and Morals.—The academy has already propoſed the following queſtions in Phyſic. The prize for each will be proportioned to the utility of the diſſertation, at the diſcretion of the preſident and director, but if approved of will be at leaſt 100 rix dollars:

I. Quæ-

I. Quænam ſit ſpecies dyſenteriæ Indis propriæ, quomodo ea ab Europea differt, quibuſve remediis ſit arcenda ?

II. Cur obſtructio viſcerum, appellata *Koek*, ſit adeo communis Batavis, et quomodo hujus progreſſus ſiſtatur ?

III. Quinam ſint fructus, herbæ atque radices medicatæ indigenæ, de quarum virtute adhuc nil conſtabat ?

The diſſertations on theſe ſubjects are to be written in Dutch, French, or Latin, and addreſſed to the preſident and director.

---

Dr. Bland, phyſician in London, has lately communicated to the Royal Society ſome intereſting obſervations, founded on the midwifery reports of the Weſtminſter General Diſpenſary, and tending to aſcertain the number of accidents or deaths that happen in conſequence of parturition, and the proportion of male to female children. The ingenious author has likewiſe attempted to determine the chance of life at different periods, from infancy to twenty-ſix years of age, and the proportion of natives to the reſt of the inhabitants of London. Theſe enquiries afford a melancholy proof of the mortality that prevails amongſt the children of the poorer claſs of people in this metropolis, ſince it appears, that five out of twelve die before they have attained the age of two years, and that only one half of all that are born live to be four years old. The ſmall proportion of natives to the reſt of the inhabitants of London is likewiſe another ſtriking reſult of Dr. Bland's reſearches, and proves, at how great an expence to the country the population of this great city is ſupported.

---

Mr. Andrews, jun. ſurgeon at Bampton in Oxfordſhire, in a letter to Dr. William Hunter, F. R. S. lately read at the Royal Society, gives an account of a remarkable operation performed on a broken arm in a young man of eighteen, by trade a maltſter. The fractured end of the bone, with part of the capſular ligament adhering to it, was forced through the pectoralis major and integuments, ſo that about three inches of the bone protruded through the wound. When Mr. Andrews and his father ſaw the patient, they recollected the caſe deſcribed by Mr. White in the *Phil. Tranſ.* Vol. LI. and determined mined to imitate the operation there ſpoken of, by ſawing off the protuding portion of the bone, rather than attempt a reduction of it, as this would have required conſiderable tenſion and an enlargement of the wound, which might have endangered the axillary artery. Beſides, from the long expoſure of the bone to the air, a conſiderable exfoliation muſt have been expected.—A ſpatula being placed between the bone and the ſkin to guard it againſt the action of the ſaw, the protruding part of the bone was ſawed off, and the remainder properly replaced. The arm was confined cloſe to the ſide, with the elbow bent. —In the courſe of the treatment, two or three abſceſſes formed at different parts of the arm and breaſt, which were opened and healed without difficulty. At the end of the ſeventh week the renovated bone had acquired conſiderable ſtrength, ſo as to allow of motion to the arm. The patient has now the perfect uſe of it, and it is not at all ſhorter than his other arm.

---

Dr. Hunter of York, F. R. S. has lately experienced the efficacy of a vapour bath, in a caſe of hydrocephalus internus. The diſeaſe, previous to the trial of this remedy, is ſaid to have have been confiderably advanced, the fight of the right eye having been much affected, and the ufe of the child's limbs almoft totally loft. By perfevering in the ufe of this bath a fhort time, the patient was cured.—In another cafe of this kind, a refpectable phyfician in London has feen favourable effects take place from the ufe of calomel. The child took a grain of it daily; when the mouth began to be affected, it was difcontinued for a few days, and afterwards had recourfe to again. This courfe was purfued, till the patient had taken about thirty grains of calomel. During all this time the progrefs of the difeafe feemed at leaft to be fufpended by the remedy, as the child's head meafured eighteen inches in circumference previous to the exhibition of the mercury, and there was no apparent increafe in its bulk during the mercurial courfe. But at length the parents of the child becoming unwilling to try it any longer, it was difcontinued, and fince that time the head has gradually increafed in fize, meafuring now about twenty-two inches in circumference.

---

Mr. Henry Smeathman, an ingenious naturalift, intends foon to publifh an account of his travels travels in Africa and the Weſt Indies, in two volumes quarto. The continent of Africa and its productions are little known to Europeans, and have long been objects of curioſity to the lovers of natural hiſtory, ſome few of whom, it ſeems, ſuggeſted the plan of a voyage to that quarter of the globe, and enabled the author to embark with the neceſſary apparatus for making collections and obſervations in thoſe countries, where he reſided near eight years, promoting, as far as circumſtances would permit, the chief object of his travels.

Mr. Smeathman, however, did not confine his views merely to natural hiſtory, but likewiſe ſtudied the diſeaſes of Africa, which he means to deſcribe in his work, together with the different modes of treatment practiſed by the natives and by Europeans.

---

At the ſale of the late Mr. Blackall's anatomical preparations on Monday, May 1, the double uterus and vagina deſcribed by Dr. Purcell of Dublin in the 64th volume of the *Philoſ. Tranſ.* was purchaſed by Mr. John Hunter for fifty guineas.—In the above collection, among ſeveral preparations of diſeaſes, the following, on account count of their ſingularity, ſeem worthy of being recorded. 1. *A tumour compreſſing the oeſophagus.* —This was taken from a man who died in St. Bartholomew's Hoſpital in Dec. 1779, and whoſe deglutition was prevented by a large tumour ſituated in the lungs, below the bifurcation of the trachea. During life, a tumour (ſeemingly ſcrophulous) appeared on the left ſide of the neck above the level of the larynx. When cut into after death, it was found to contain a fœtid, conſiſtent matter. The pharynx and upper part of the oeſophagus were overſpread with a malignant (apparently cancerous) ulceration, which had deſtroyed in one point the ſubſtance of the oeſophagus, and there the cavity of the tumour in which the matter before-mentioned was lodged, communicated with the oeſophagus. 2. *A diſeaſed hand.*—This was amputated on account of a caries of the bones of the middle finger, and an hæmorrhage which could not be reſtrained. On diſſection, all the arteries of the part appeared diſeaſed, dilated, and aneuriſmal. 3. *A communication between the ſtomach and gall bladder.*—This was taken from a man who died of an aſcites, after being tapped. On diſſection, a conſiderable quantity of blood was found in the abdomen. 4. *A communication between the*

*trachea and the pulmonary artery.*—The ſubject of this diſeaſe died in St. Bartholomew's hoſpital in 1780, by a ſudden diſcharge of blood, ſeemingly arterial, from the mouth. On diſſection, a large communication appeared to have taken place between the right branch of the pulmonary artery, and the ſame branch of the trachea.

5. *A diſeaſe of the urethra and bladder.*—This preparation was taken from a man whoſe complaints originated from an obſtructed urethra; for ſome time before his death, his urine, inſtead of paſſing through the penis, was diſcharged through five or ſix fiſtulæ in the ſcrotum, the patient experiencing great pain about the bladder and rectum. On diſſection, oppoſite to the fiſtulæ in the ſcrotum, an obſtruction was diſcovered in the urethra, occaſioned primarily by a ſtricture of that canal, but completed by ſeven or eight calculi which had been cauſed by it, and which were lodged immediately behind it. The bladder, which was greatly thickened, was filled with mucus, and contained ſix calculi. Behind the bladder was a maſs of thickened parts connecting it with the rectum, and a communication between the bladder and rectum large enough to admit a bougie.

At a public meeting of the Academy of Surgery at Paris, on the 26th of April, the gold medal of 500 livres value was adjudged to the celebrated Profeſſor Camper for the beſt diſſertation on the prize queſtion announced laſt year; after which were read the elogy of the late M. Diſdier, by M. Louis; ſome reflexions on the exfoliation of bones by M. Fabre; remarks on abſceſſes in the ſubſtance of the tongue by M. Petibeau; and a diſſertation on the locked jaw by M. Sabatier.

